# Genetic structure of *Trypanosoma congolense* “forest type” circulating in domestic animals and tsetse flies in the South-West region of Cameroon

**DOI:** 10.1051/parasite/2017052

**Published:** 2017-12-19

**Authors:** Pythagore Soubgwi Fogue, Flobert Njiokou, Gustave Simo

**Affiliations:** 1 Molecular Parasitology and Entomology Unit, Department of Biochemistry, Faculty of Science, University of Dschang, Dschang Cameroon; 2 Department of Animal Biology and Physiology, Faculty of Science, University of Yaoundé I, Yaoundé Cameroon

**Keywords:** *Trypanosoma congolense* “forest type”, microsatellite markers, tsetse fly, domestic animals, population genetics

## Abstract

Despite the economic impact of trypanosome infections, few investigations have been undertaken on the population genetics and transmission dynamics of animal trypanosomes. In this study, microsatellite markers were used to investigate the population genetics of *Trypanosoma congolense* “forest type”, with the ultimate goal of understanding its transmission dynamics between tsetse flies and domestic animals. Blood samples were collected from pigs, sheep, goats and dogs in five villages in Fontem, South-West region of Cameroon. In these villages, tsetse were captured, dissected and their mid-guts collected. DNA was extracted from blood and tsetse mid-guts and specific primers were used to identify *T. congolense* “forest type”. All positive samples were genetically characterized with seven microsatellite markers. Genetic analyses were performed on samples showing single infections of *T. congolense* “forest type”. Of the 299 blood samples, 137 (46%) were infected by *T. congolense* “forest type”. About 3% (54/1596) of tsetse fly mid-guts were infected by *T. congolense* “forest type”. Of 182 samples with *T. congolense* “forest type”, 52 were excluded from the genetic analysis. The genetic analysis on the 130 remaining samples revealed polymorphism within and between subpopulations of the target trypanosome. The dendrogram of genetic similarities was subdivided into two clusters and three sub-clusters, indicating one major and several minor genotypes of *T. congolense* “forest type” in tsetse and domestic animals. The low *F_ST_*values suggest low genetic differentiation and no sub-structuration within subpopulations. The same *T. congolense* genotypes appear to circulate in tsetse and domestic animals.

## Abstract (fr)

Malgré l'impact économique des infections trypanosomiennes, peu d'investigations ont été entreprises sur la génétique des populations et la dynamique de transmission des trypanosomes animaux. Dans cette étude, des marqueurs microsatellites ont été utilisés pour étudier la génétique des populations de *Trypanosoma congolense* « type forêt » dans le but de comprendre sa dynamique de transmission entre les mouches tsé-tsé et les animaux domestiques. Des échantillons de sang ont été prélevés chez des porcs, des moutons, des chèvres et des chiens de cinq villages de Fontem dans la région Sud-Ouest du Cameroun. Dans ces villages, les mouches tsé-tsé ont été capturées, disséquées et leurs intestins moyens recueillis. L'ADN a été extrait d'échantillons de sang et d'intestins des mouches tsé-tsé et des amorces spécifiques ont été utilisées pour identifier *T. congolense* « type forêt ». Tous les échantillons positifs ont été génétiquement caractérisés avec sept marqueurs microsatellitaires. Des analyses génétiques ont été effectuées sur des échantillons présentant des infections simples de *T. congolense* « type forêt ». Sur les 299 échantillons de sang, 137 (46 %) étaient infectés par *T. congolense* « type forêt ». Environ 3 % (54/1596) des intestins de mouches tsé-tsé étaient infectés par *T. congolense* « type forêt ». Sur 182 échantillons porteurs de *T. congolense* « type forêt », 52 ont été exclus des analyses génétiques. Sur les 130 échantillons restants, les analyses génétiques ont révélé un polymorphisme au sein et entre les sous-populations du trypanosome cible. Le dendrogramme de similarités génétiques a été subdivisé en deux groupes et trois sous-groupes, indiquant ainsi un génotype majeur et plusieurs génotypes mineurs de *T. congolense* « type forêt » chez les tsé-tsé et les animaux domestiques. Les faibles valeurs de *F_ST_*impliquent une faible différenciation génétique et aucune sous-structuration des sous-populations. Les mêmes génotypes de *T. congolense* semblent circuler chez les tsé-tsé et les animaux domestiques.

## Introduction

African trypanosomiases are parasitic infections caused by pathogenic protozoa of the Trypanosomatidae family. Trypanosomes are cyclically transmitted to animals by tsetse flies. In 37 sub-Saharan African countries, African animal trypanosomiases (AATs) represent an important factor that limits cattle breeding in an area of about 9 million km^2^. AATs cause high mortality, abortion, significant reductions in milk and meat production, and delay in sexual maturity, along with low calving rates and loss of draught power. These diseases are associated with considerable losses in livestock production [25]. The total losses due to AATs are estimated at about USD 4.75 billion per year [27].

The main pathogenic trypanosomes of livestock are *Trypanosoma congolense*, *Trypanosoma vivax* and *Trypanosoma brucei brucei* [3]. In addition to *T. congolense*, trypanosomes of the subgenus *Nannomonas* contain also *T. simiae* which is found in a wide range of ungulate hosts. The use of biochemical and molecular methods subdivided trypanosomes of the subgenus *Nannomonas* into two clades [11]. The first clade, or *T. congolense* clade, contains *T. congolense* associated with savannah and forest and another distinct group or *T. congolense* kilifi, which was found around the Kenyan coast [9,16,21]. The savannah group was further subdivided into East and West Africa [10]. The second clade, or *T. simiae* clade, contains *T. simiae*, *T. godfreyi-*like, *T. simiae* tsavo and *T. godfreyi* [11].

In sub-Saharan Africa, *T. congolense* has been shown to be the most prevalent trypanosome species infecting cattle [19,27,28,41,42]. Controlling *T. congolense* infections requires investigations aiming to better understand the population genetics of trypanosomes. Such investigations could help to comprehend the spread of genotypes responsible for specific pathophysiological traits such as drug resistance or different clinical manifestations. Previous studies have shown that isolates of *T. congolense* exhibit different clinical phenotypes in the infected hosts [23]. For instance, the virulent isolates of *T. congolense* induce acute disease and high mortality, while the less virulent strains cause benign and chronic infections [24]. Although the reasons explaining these pathophysiological differences are not well understood, the genetic variability between strains could play an important role. In this light, investigations on the genetic variability of trypanosome isolates could help to decrypt the genetic basis of the pathophysiological differences occurring in infected hosts, as well as the transmission dynamics of trypanosomes.

During the last few decades, several genetic markers including Amplified Fragment Length Polymorphism (AFLP) [1,35], Randomly Amplified DNA Polymorphism (RADP) [15], minisatellite [20] and microsatellite DNA sequences [4,17,27] have been used for the genetic characterization of trypanosomes. Amongst these markers, the microsatellite markers have been widely used because of their high level of polymorphism, their reproducibility, their specificity and sensitivity. In Human African trypanosomiasis (HAT), these markers enabled correlations to be drawn between the genetic variability of *T. brucei* and different epidemiological pictures of HAT in west and central Africa [17,33,36,37]. In the Fontem HAT focus, microsatellite markers revealed specific genotypes of *T. brucei* in tsetse flies and different animal species. They also revealed common genotypes circulating in both tsetse flies and different animal species [39], highlighting the need to understand the transmission dynamics of trypanosome genotypes between different hosts. In AAT, investigations on the population genetics of the forest and savannah types of *T. congolense* revealed predominant clonal reproduction within *T. congolense* “forest type” [38,40], and more likely sexual reproduction in *T. congolense* “savannah type” [27]. Microsatellite markers also enabled researchers to identify different genotypes of *T. congolense* in tsetse flies and different animal species [27,38,40]. However, most studies compared strains or isolates from animals, or circulating either in animals or tsetse flies, or of different tsetse-infested areas. The results generated from these studies cannot explain the transmission dynamics of trypanosomes in a particular area. It is in this light that investigations were recently undertaken on the genetic polymorphism of *T. congolense* circulating in tsetse flies and domestic animals of Fontem in southern Cameroon [38,40]. Since these studies analyze data generated on animals or tsetse flies separately, they cannot be used to understand the circulation of trypanosome genotypes. Until now, no studies have investigated the genetic differentiation between *T. congolense* isolates circulating both in tsetse flies and different animal species of the same tsetse-infested area. Such investigations could help to understand the transmission dynamics as well as the circulation of trypanosome genotypes between hosts (tsetse flies and different animal species) or villages. In this context, reviewing and reanalyzing published data of the same tsetse-infested area could enable us to understand the transmission dynamics and the circulation of trypanosome genotypes, and also to improve our knowledge on the genetics of trypanosomes.

In this study, single infections of *T. congolense* “forest type” were retrieved from data of two studies published by Simo *et al*. [38,40]. The retrieved data were subsequently reanalyzed in order to understand the transmission dynamics as well as the circulation of *T. congolense* genotypes between hosts (tsetse flies and different domestic animals) or different villages of Fontem.

## Material and methods

### Ethics statement

For this study, verbal authorizations were obtained from inhabitants, and from traditional and administrative authorities in each village before beginning the study. Thereafter, verbal consent was obtained from each owner before sample collection.

### Study area

This study was carried out in the Fontem (05°40'12”N, 09°55'33”E) region located in the Lebialem, Manyu and Koupe-Manengouba Divisions of Cameroon. This is a forested region with a tropical humid climate. The topography is made up of hills and valleys through which several high-speed rivers flow [2]. The main activities of inhabitants are agriculture, palm oil extraction, animal husbandry and poultry farming at a small scale. Previous studies on African trypanosomiases have revealed several trypanosome species and subspecies in tsetse flies and animals in this region [29,31,34]. Domestic animals such as dogs, pigs, sheep, and goats are bred by most inhabitants for family incomes. *Glossina palpalis palpalis* has been reported as the main vector of trypanosomes in this locality [26].

### Sample collection

Domestic animals were sampled in five villages (Agong, Bechati, Besali, Folepi and Menji) of Fontem in July 2006 and June 2007. Before sampling, the objective of the study was explained to local authorities and inhabitants of each village. After their approval, inhabitants were asked to catch and/or keep their domestic animals. All domestic animals that had spent at least 3 months in the study zone were selected. All details concerning the animals sampled and the blood collection procedures were described by Simo *et al*. [38,40].

Tsetse flies were sampled during two entomological surveys conducted in November 2006 and April 2007. These entomological surveys were performed in the same five villages (Agong, Bechati, Besali, Folepi and Menji) where domestic animals were sampled. During each survey, pyramidal traps [13] were set in tsetse fly-favorable biotopes. Details regarding the entomological surveys were described by Simo *et al*. [40].

### DNA extraction

DNA was extracted from blood samples using the “DNeasy Tissue kit” (Qiagen) as described by Simo *et al*. [37]. Initially, 1 ml of blood was mixed with an equal volume of nuclease-free water. The mixture was homogenized for 10 minutes and thereafter, centrifuged at 14 000 rpm for 10 minutes. The supernatant was discarded and the pellet containing parasites was re-suspended in 200 μl of phosphate-buffered saline (PBS). DNA extraction was carried out on the re-suspended pellet, following the instructions of the manufacturer. The DNA extract was used directly for PCR or stored at −20°C until use.

For the tsetse fly mid-guts, the Chelex method was used to extract DNA [43]. Briefly, the alcohol used to preserve tsetse fly mid-guts was evaporated for 60 minutes at 80°C. Thereafter, 300 μl of Chelex 5% was introduced in each microtube and the mixture vortexed for 10 minutes. Each microtube was subsequently incubated at 56°C for one hour, and then at 100°C for 30 minutes. These microtubes were centrifuged at 14 000 rpm for 10 minutes and the supernatant (DNA extract) was collected and stored at −20°C until use.

### Identification of *T. congolense* “forest type”

For this identification, specific primers (TCF1/2) for *T. congolense* “forest type” were used [22]. The amplification reactions were carried out as described by Herder *et al*. [14]. All samples positive for *T. congolense* “forest type” were selected and subjected to subsequent analyses.

### Genetic characterization of *T. congolense* “forest type”-positive samples

The samples positive for *T. congolense* originating from tsetse flies and domestic animals were genetically characterized at seven microsatellite loci, as previously described by Morrison *et al*. [27]. For each sample, two PCR rounds were performed as described by Simo *et al.* [38,40]. The amplified products of the nested PCR were checked by electrophoresis on 2% agarose gel and the allele sizes were resolved by electrophoresis in 10% non-denaturing acrylamide gels. A sample containing more than two alleles (two DNA fragments) or with at least three alleles (three DNA fragments) for at least one microsatellite marker was considered here as having multiple genotypes because *T. congolense* is a diploid organism and must have one allele (homozygote) or two alleles (heterozygote) after the resolution of the amplified products of each microsatellite locus.

### Genetic analyses

For these analyses, samples with multiple genotypes or showing more than two alleles were excluded. Therefore, only single genotypes or samples showing no more than two alleles were taken into account for the analysis of population genetic structure. Due to the low amplification rate of the TCM3 and TCM5 markers, the data generated for these two microsatellite markers were not considered for genetic analyses. In addition, data for TCM4 were also not considered because no polymorphism was observed. Therefore, only data generated by four microsatellite markers were used for population genetics studies. The genetic structure within and between subpopulations of *T. congolense* “forest type” was assessed through Wright's F-statistics [45]. For this assessment, *F_ST_* values were evaluated. *F_ST_*is a measure of deviation from random distribution of individuals between subpopulations (and thus, population differentiation). *F_ST_* measures the genetic differentiation between subpopulations. Here, we considered as a subpopulation all samples of *T. congolense* “forest type” originating from the same host (goat, pig, dog, sheep and tsetse) or the same village (Agong, Bechati, Besali, Folepi and Menji). *F_ST_*is a convenient measure of differentiation among the different subpopulations of a data set. The *F_ST_*estimator is around 0 under the null hypothesis of random distribution of genotypes across subpopulations. The estimator displays positive values, up to 1, in case of genetic differences. Wright's F-statistics were estimated using Weir and Cockerham's unbiased estimators [44] in Fstat 2.9.4 software [12]. For *F_ST_*, the estimator was *Ө* and its significance was tested through 10,000 permutations of individuals between subpopulations.

To get an overall idea of individual distribution across the hosts, unrooted NJTREEs were computed with MEGA 3.1 software [18] using the Cavalli-Sforza and Edwards [5] chord distances matrix, which was computed in MSA software [7] and formatted in PHYLIP 3.69 software [8]. The allelic richness was estimated with *Fstat* 2.9.4 software. The test was performed through 10,000 permutations within subsamples.

## Results

For this study, 299 domestic animals and 2695 *Glossina palpalis palpalis* were sampled in five villages (Agong, Bechati, Besali, Folepi and Menji) of Fontem. The 299 domestic animals included 173 pigs, 60 sheep, 48 goats and 18 dogs (Table 1). Tables 1 and 2 contain details regarding sample distribution according to villages and different hosts.

**Table 1:** Infection rates with *T. congolense* “forest type” according to villages and domestic animal species

Villages	Number of each animal species (%)				Total
	Pig	Sheep	Goat	Dog	
Agong	9 (11.11)	8 (12.5)	2 (50.0)	0 (0)	19 (15.79)
Bechati	39 (58.97)	11 (54.54)	37 (29.72)	3 (0)	90 (44.44)
Besali	24 (41.66)	33 (24.24)	7 (28.57)	3 (0)	67 (29.85)
Folepi	19 (68.42)	6 (16.66)	0 (0)	12 (16.66)	37 (43.24)
Menji	82 (65.85)	0 (0)	4 (100)	0 (0)	86 (67.44)
Total	173 (58.38)	58 (27.58)	50 (36.00)	18 (11.11)	299 (45.92)

(): Infection rate with *T. congolense* “forest type”

**Table 2:** Tsetse fly mid-gut infections with *T. congolense* “forest type” according to villages

Villages	Number of tsetse captured	NT (%)
Agong	25	22 (4.54)
Bechati	516	345 (3.48)
Besali	40	9 (0)
Folepi	1909	1045 (3.25)
Menji	205	175 (4.00)
Total	2695	1596 (3.38)

NT: number of tsetse mid-guts examined; (): infection rate with *T. congolense* “forest type”

### Molecular identification of *T. congolense* “forest type” in domestic animals and tsetse fly mid-guts

For the 299 domestic animals, the specific PCR targeting a multi-copy repeat sequence of *T. congolense* “forest type” revealed 137 domestic animals (45.92%) infected by this trypanosome (Table 1). Among the 137 infected animals, nine co-infections of *T. congolense* “forest type” and *T. congolense* “savannah type” were observed. These nine samples were excluded for genetic characterization because the primers used for the microsatellite sequences amplify *T. congolense* “forest type” as well as *T. congolense* “savannah type”. Therefore, 128 instead of 137 *T. congolense* “forest type”-positive samples from domestic animals were characterized genetically with microsatellite markers.

Of the 2,695 *G. p. palpalis* that were captured, 1,596 were dissected and their mid-guts collected. On these 1,596 mid-guts, 54 were infected by *T. congolense* “forest type” (3.38%) (Table 2). The 54 infected mid-gut samples were also subjected to genetic characterization with the same microsatellite markers used for samples from domestic animals.

### Genetic characterization of *T. congolense* “forest type” of tsetse flies and domestic animals

For this characterization, seven microsatellite markers were used. No samples were amplified by TCM3, while TCM5 amplified less than 20% of *T. congolense* “forest type”-positive samples. Results of these two markers were not considered during genetic analyses. The remaining five markers (TCM1, TCM2, TCM4, TCM6 and TCM7) generated good results with amplification rates above 80%. However, TCM4 generated no polymorphism (presence of single allele of 152 bp) for all samples and consequently was not also considered during subsequent analyses. Excluding results for three microsatellite markers (TCM3, TCM4 and TCM5) enabled us to perform the genetic analyses on data generated at four microsatellite loci.

Of the 182 *T. congolense* “forest type”-positive samples (128 from domestic animals and 54 from tsetse flies), 36 different alleles were identified: 30 and 6 in single and mixed genotypes, respectively. Specific alleles such as 136 bp for TCM2, 138 bp and 173 bp for TCM1, and 208 bp, 218 bp, 233 bp for TCM7 were observed only in mixed genotypes (Table A1).

Thirteen *T. congolense* “forest type”-positive samples were not amplified at the four microsatellite markers considered here. They were therefore excluded during genetic analyses. Of the 169 remaining samples, 130 were single genotypes, whereas 39 were mixed genotypes of *T. congolense* “forest type”. Thirty-six mixed genotypes were from domestic animals and three from tsetse flies. The genetic analyses were performed on samples containing both single genotypes and alleles at the four microsatellite loci considered in this study. With the exclusion of 13 samples showing incomplete data at some of the four loci, together with 39 samples with mixed genotypes, 52 *T. congolense* “forest type”-positive samples were not ultimately subjected to genetic analysis. A total of 130 samples (43 from tsetse flies and 87 from domestic animals) with single genotypes of *T. congolense* “forest type” were finally considered for population genetics studies. Table A1 contains details on each sample as well as the allele size at each locus.

### Allelic frequencies

**Figure 1:**
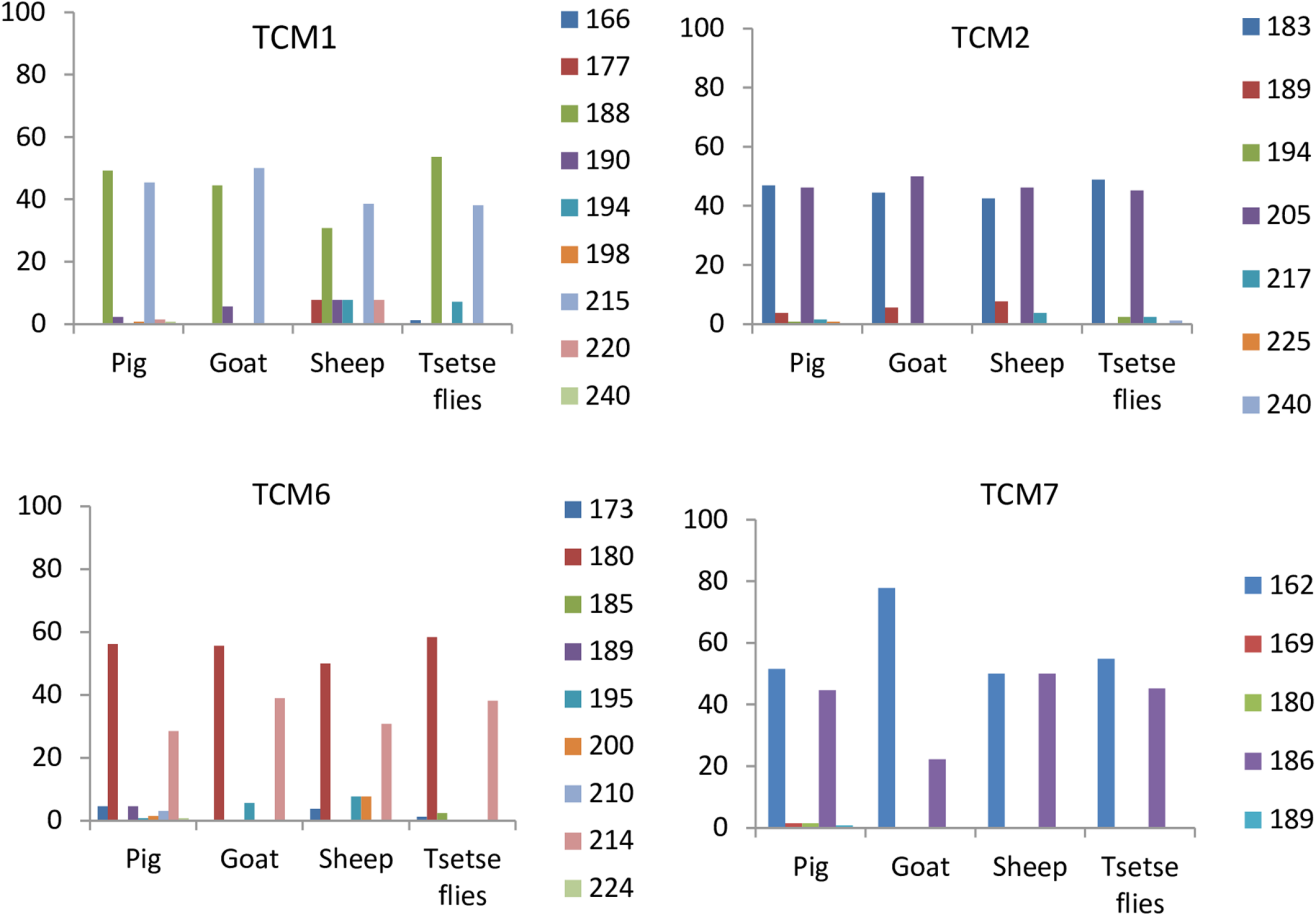
Allelic frequency at each locus by host.

High allelic frequencies above 40% were observed for some alleles of *T. congolense* “forest type” circulating both in tsetse flies and all domestic animals (Figure 1). Considered as belonging to major genotypes of *T. congolense* “forest type”, these alleles include for instance 188 bp and 215 bp of TMC1, 183 bp and 205 bp of TCM2, 180 bp of TCM6, and 162 bp and 186 bp of TCM7. Other alleles with very low allelic frequencies (below 20%) were observed. These alleles can be considered as minor alleles belonging to minor genotypes of *T. congolense* “forest type”. They include 189 bp and 217 bp of TCM2, 190 bp, 194 bp and 198 bp of TCM1, 195 bp and 200 bp of TCM6, and 169 bp and 180 bp of TCM7. Considering single genotypes only, it was found that 15 alleles were observed in *T. congolense* “forest type” identified in flies, against 27 in animals. The number of alleles circulating in animals is 1.8 times higher (27/15) than the value obtained for parasites found in tsetse flies.

### Allelic richness and genetic diversity index

The values for the allelic richness and genetic diversity indexes can be found in Table 3. The allelic richness calculates the number of alleles per locus by taking into account the number of samples. Its value was 1.469 for *T. congolense* “forest type” circulating in animals and 1.478 for parasite identified in tsetse flies. No significant difference (*p* *=* *0.936*) was observed between these values. The genetic diversity index evaluates the degree of varieties of genes in a given population. Its value was 0.569 for *T. congolense* “forest type” circulating in animals and 0.540 for parasites identified in tsetse flies. No significant difference (*p* *=* *0.148*) was observed between these values. With the overall value of 0.554, some variation in the genetic diversity indexes was observed across loci (Table 4).

**Table 3:** Allelic richness and genetic diversity index for *T. congolense* “forest type” circulating in domestic animals and tsetse flies.

	Loci	Domestic animals	Tsetse flies
Allele per locus	TCM1	177*, 188, 190*, 194, 198*, 215, 220*, 240*	166**, 188, 194, 215
	TCM2	183, 190*, 194, 205, 217, 225*	183, 194, 205, 217, 240**
	TCM6	173, 180, 189*, 195*,200*, 210*, 214, 224*	173, 180, 185**, 214
	TCM7	162, 169*, 180*, 186, 190*	162, 186
Total alleles		27	15
Allelic richness		1.469	1.478
*p*		0.936	
Genetic diversity		0.569	0.540
*p*		0.148	

*: alleles found only in *T. congolense* “forest type” circulating in domestic animals; **: alleles found only in *T. congolense* “forest type” circulating in tsetse flies.

**Table 4:** Genetic diversity index at each locus

Locus	TCM1	TCM2	TCM6	TCM7	Overall
N	9	7	9	5	30
Ho	0.930	0.984	0.618	0.868	0.850
Hs	0.629	0.576	0.509	0.502	0.554

N: Number of alleles; Ho: Observed genetic diversity; Hs: Expected genetic diversity

### Neighbor-joining analysis

**Figure 2:**
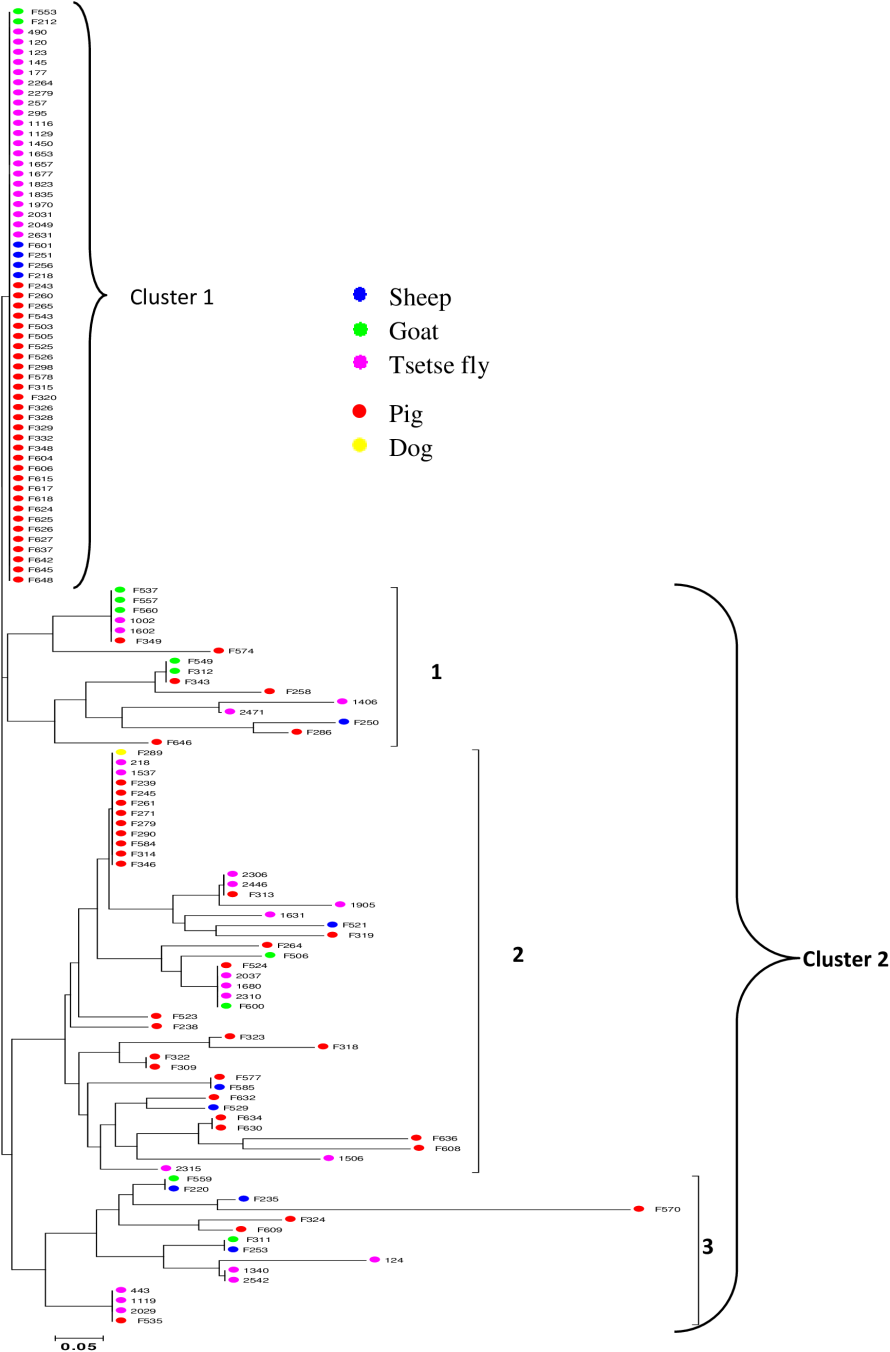
NJ Tree based on Cavalli-Sforza and Edwards chord distance matrix of *T. congolense* “forest type” circulating in tsetse flies and domestic animals of Fontem.

The dendrogram illustrating the genetic similarities between *T. congolense* “forest type” strains shows low genetic distances between most samples. It also shows the heterogeneous distribution of *T. congolense* “forest type” genotypes, indicating a lack of genetic clustering by hosts or villages. For the 130 samples considered for the population genetics studies, 42 distinct multilocus genotypes were observed (Figure 2). These genotypes can be subdivided into two main clusters. The first cluster with one genotype of 57 samples can be considered as the major genotype. Of these 57 samples, 89.47% (30 from pigs and 21 from tsetse flies) were from pigs and tsetse flies. The six remaining samples were from goats and sheep (Figure 2). The second cluster with 40 genotypes and 73 samples can be subdivided into three sub-clusters. Samples belonging to these sub-clusters probably harbor minor genotypes of *T. congolense* “forest type”. Samples from goats (5/11) are mainly found in sub-cluster 1. Sub-cluster 2 contains samples from pigs (59.52%) and tsetse flies (26.19%). Samples from sheep are predominantly found in sub-clusters 2 and 3. The genotypes circulating in pigs are found in various clusters and sub-clusters (Figure 2).

### Genetic differentiation between *T. congolense* “forest type” subpopulations

The *F_ST_* values were very low and not significant between subpopulations of *T. congolense* “forest type” according to villages (Table 5). Despite the significant difference between *T. congolense* “forest type” subpopulations of tsetse flies and sheep (*F_ST_* = 0.01: *p* = 0.005), the *F_ST_* values were generally very low and not significant between hosts (Table 6). These results indicate a lack of genetic differentiation between *T. congolense* “forest type” subpopulations according to villages or different hosts (tsetse flies and different animal species). They therefore reflect the low genetic distances between strains of *T. congolense* “forest type” circulating in tsetse and domestic animals of Fontem.

**Table 5:** *F_ST_*values between subpopulations of *T. congolense* “forest type” by village

	Bechati	Besali	Folepi	Menji
Agong	-0.0189	-0.0181	-0.0196	-0.0134
Bechati		-0.0002	-0.0003	0.0034
Besali			0.0038	0.0034
Folepi				0.0022

**Table 6:** *F_ST_* values between subpopulations of *T. congolense* “forest type” by trypanosome host

	Goat	Sheep	Tsetse fly
Pig	0.0146	0.0041	0.0031
Goat		0.0132	0.0142
Sheep			0.0123*

*: significant *p* value.

## Discussion

This study on the genetic structure of *T. congolense* “forest type” has shown some genetic polymorphism between *T. congolense* “forest type” circulating in tsetse flies and domestic animals of different villages of Fontem. Samples showing more than two alleles or samples with mixed genotypes of *T. congolense* “forest type” were excluded from population genetics studies because the genetic information for each individual trypanosome could not be obtained. Some alleles involved in these infections (probably minor genotypes) were not taken into account and consequently, the real genetic variability of *T. congolense* “forest type” was underestimated in the studied areas. Non-consideration of wild animals, known as hosts for trypanosomes and as a source of blood meals for tsetse flies [30], is another argument strengthening the underestimation of the genetic variability of *T. congolense* “forest type”. Irrespective of the microsatellite marker used, the majority of alleles identified in tsetse flies and domestic animals were selected for population genetics studies. The resulting genotypes are probably the major ones circulating in the studies areas. The identification of *T. congolense* “forest type” in tsetse flies and domestic animals confirms previous results [26,29,34].

For population genetics studies performed with data generated at four microsatellites loci, 27 alleles were identified in domestic animals and only 15 in tsetse flies. These results indicate a high level of genetic variability within strains circulating in animals, compared to those found in tsetse flies. They are in line with previous observations reporting that the full level of trypanosome diversity is only apparent when the parasites circulating in tsetse flies are examined [20,40]. The discrepancy between the number of alleles found in trypanosomes circulating in animals and that of tsetse flies could partially be explained by the low life expectancy of tsetse flies (cannot live for more than four months) compared to animals. In this context, tsetse flies cannot accumulate infections in the mid-guts like mammals, which can be infected successively by tsetse flies harboring different *T. congolense* strains. Another reason explaining this discrepancy is the bottleneck observed during the establishment and migration of trypanosomes in tsetse fly. This bottleneck enables few parasites to complete their developmental cycle in tsetse flies while the tsetse immune response kills most trypanosomes during the first days after a blood meal.

The absence of significant differences between values for allelic richness as well as the genetic diversity index suggests that the genetic variability between strains of *T. congolense* “forest type” was not affected by the difference observed in the number of alleles identified between trypanosomes circulating in tsetse flies and domestic animals.

Comparing the number of alleles (12 out of 30 alleles) identified in different hosts (tsetse flies and domestic animals) with those observed for *T. brucei* s.l. (12 out of 70 alleles) in the same villages [38], it appears that a high number of identical genotypes of *T. congolense* circulate both in tsetse and domestic animals. This is strengthened by the clustering of several genotypes from animals with those from tsetse flies (Figure 2). The identification of several genotypes in the same host indicates the heterogeneous nature of *T. congolense* strains circulating in villages of Fontem. This observation is highlighted by the strong heterogeneous distribution of strains without any real sub-clustering by host or village (Figure 2).

Although it is surprising to see that strains of two populations (originating from tsetse fly and domestic animals) of *T. congolense* “forest type” can be subdivided into two main clusters, separate analyses of each of these populations did not reveal such results [38,40]. The two main clusters identified here probably result from the mixture of two populations of *T. congolense* “forest type” that circulate in tsetse flies and mammalian hosts. Analysis of such a mixture enabled a better understanding of the genetic diversity of trypanosomes as well as the genetic structure of these parasites. The genotypes belonging to the same cluster or sub-cluster of the dendrogram can be considered as closely related strains or closely related genotypes. The identification of several genotypes within the same cluster highlights some genetic polymorphisms between strains of *T. congolense* “forest type” as reported previously by Simo *et al*. [38,40]. No cluster or sub-cluster can be linked to a specific host of trypanosomes. Cluster 1 with 57 strains originating mainly from tsetse flies and pigs can be considered a major genotype circulating in the villages of Fontem. The presence of one genotype with 57 strains indicates identical genotypes or closely related genotypes circulating between villages or different hosts (tsetse flies and different animal species). This is strengthened by high allelic frequencies for some alleles identified in almost all trypanosome hosts (Figure 1). In other clusters and sub-clusters, where genetic polymorphism is high with about 40 genotypes for 73 samples, the *T. congolense* strains were from various hosts. This high genetic polymorphism can be explained by several minor genotypes circulating in tsetse flies and different domestic animals. The very low allelic frequency is in favor of minor *T. congolense* genotypes circulating in different villages (Figure 1). Results of mixed infections highlight the co-existence of minor and major *T. congolense* genotypes within a host. Some genotypes identified in tsetse flies and not in animals, or in one animal species but not in another, can be considered as minor genotypes that could be specific to a particular host. Such genotypes are generally characterized by very low allelic frequencies. The minor genotypes identified in tsetse were probably from wild animals not analyzed in this study. Moreover, given the high virulence of some *T. congolense* strains, it is important to point out that some genotypes that seem to be specific to tsetse fly or appearing only in tsetse fly could induce rapid death in most infected mammalian hosts that were sampled in this study. The minor genotypes identified in animals but not in tsetse flies could be explained by the bottleneck which is a demographic process in which a population of trypanosomes can suffer from a sharp decline of effective size [6]. In tsetse fly mid-guts, this bottleneck could significantly reduce the number of a particular strain to a point that it becomes undetectable during PCR reactions [32]. Such strains could continue maturation and later be transmitted to animals during tsetse fly blood meals [37]. Some minor genotypes found in animals could result from their circulation in a minority, not because they are specific to certain hosts, but due to their virulence and high pathogenicity that probably lead to rapid death of some infected animal species.

The very low values of *F_ST_* between villages with no statistical significance indicate an absence of sub-structuration by village. They suggest identical genotypes or closely related genotypes circulating in tsetse fly and different animals or between the villages of Fontem. This is highlighted by samples of various villages clustering together in different clusters and sub-clusters of the dendrogram (Figure 2). The very low values of *F_ST_* can also be explained by the fact that sampling villages such as Agong, Bechati, Besali and Folepi are neighboring villages probably with similar trypanosomes and tsetse fly populations, but also with identical transmission pictures. The circulation of identical genotypes in tsetse fly and animals of different villages may have a real impact on the transmission and the spread of specific traits like resistant or pathogenic strains. Our results do not corroborate those obtained for *T. brucei* s.l. where high and significant *F_ST_*values were observed between villages [39]. The difference between these results can be linked to trypanosome species. It appears that within the same area and for the same hosts, the transmission dynamics of trypanosome genotypes can vary according to trypanosome species or subspecies.

Between subpopulations of *T. congolense* “forest type” circulating in sheep and tsetse flies, the *F_ST_* value was very low (0.0132), but statistically significant, suggesting some genetic differences between strains circulating in tsetse flies and sheep, as previously reported for *T. brucei* [39]. Despite this significant difference, most *F_ST_* values between hosts (pigs, goats, sheep, and tsetse flies) were very low and not significant, indicating an absence of sub-structuration by host. In previous studies, MacLeod *et* *al*. [20] reported that host selection is an important determinant for the population structure of trypanosomes because a particular genotype of trypanosome can adapt and survive within a specific mammalian host. Analyzing the population genetics structure of *T. brucei* circulating in the same hosts and the same villages, Simo *et al*. [39] reported a certain level of sub-structuration. Contrary to what has been reported for *T. brucei*, our data on *T. congolense* “forest type” suggest that each host cannot be considered as a separate entity during population genetics studies. Our results suggest that for the genetic characterization of *T. congolense* “forest type”, sampling in one village or in one animal species could provide information on the population genetic structure of parasites circulating in the studied areas. Results obtained in pigs strengthen this hypothesis because trypanosome genotypes originating from pigs were widely distributed in the dendrogram. The genetic characterization of strains circulating in pigs could therefore provide information on the genetic variability of *T. congolense* “forest type” circulating in tsetse flies and animals of this region. This is in line with what has been reported for *T. brucei* [39]. Results for *F_ST_* confirm the wide distribution of strains as observed in the dendrogram. They suggest that the same genotypes or closely related genotypes circulate between villages or different hosts, without any genotype genuinely committed to a village or a particular host. This wide distribution of similar genotypes indicates the transmission of identical strains in these tsetse-infested areas. To interrupt disease transmission in such areas, similar control strategies could be implemented with the use of identical control tools like drugs and vector-control tools. With these generalized control strategies, there is a high risk for the development and spread of resistant strains in different villages. In this type of context, control operations must be followed up by continuous characterization of trypanosome isolates in order to detect emerging resistant and more pathogenic strains early that could jeopardize control efforts.

## Conclusion

The results of this study enable us to improve our knowledge on the transmission dynamics of *T. congolense* “forest type” between villages or different hosts. These results suggest no association between a genotype or closely related genotypes of *T. congolense* “forest type” to a specific host or village. They suggest that the same genotypes or genotype families circulate in tsetse flies and various domestic animals. They indicate an absence of sub-structuration between subpopulations of *T. congolense* “forest type” according to hosts or villages. The data generated here made it possible to understand the transmission dynamics as well as the spread of different genotypes of *T. congolense* “forest type”.

## Appendix A

**Table A1:** Genetic characteristics of *T. congolense* “forest type” circulating in domestic animals and tsetse flies, size of alleles at each microsatellite locus

No.	Host	Village	TCM1	TCM2	TCM6	TCM7
F600	Goat	Agong	188/215	183/205	180/180	162/162
F601	Sheep	Agong	188/215	183/205	180/214	162/186
F309	Pig	Agong	188/215	183/205	180/189	162/186
F281^a^	Goat	Bechati	188/215	183/205	180/180	162/186/218
F537	Goat	Bechati	188/215	183/205	180/214	162/162
F549	Goat	Bechati	188/215	189/205	180/214	162/186
F553	Goat	Bechati	188/215	183/205	180/214	162/186
F557	Goat	Bechati	188/215	183/205	180/214	162/162
F558 a	Goat	Bechati	188/215	183/205/240	180/180	162/162
F559	Goat	Bechati	190/215	183/205	180/214	162/186
F560	Goat	Bechati	188/215	183/205	180/214	162/162
F589 a	Goat	Bechati	173/188/240	183/205	180/180	162/186
F594 a	Goat	Bechati	138/188/215	183/205	180/180	162/162
F535	Goat	Bechati	188/188	183/205	180/214	162/186
F250	Sheep	Bechati	188/215	190/217	180/195	162/186
F251	Sheep	Bechati	188/215	183/205	180/214	162/186
F252 a	Sheep	Bechati	188/215	183/205/240	180/214	162/186
F253	Sheep	Bechati	194/220	183/205	180/214	162/186
F254 b	Sheep	Bechati	000/000	183/205	180/214	000/000
F256	Sheep	Bechati	188/215	183/205	180/214	162/186
F238	Pig	Bechati	188/215	183/205	180/224	162/186
F239	Pig	Bechati	188/215	183/205	180/180	162/186
F243	Pig	Bechati	188/215	183/205	180/214	162/186
F245	Pig	Bechati	188/215	183/205	180/180	162/186
F249 a,b	Pig	Bechati	000/000	183/205/240	185/185	162/186
F258	Pig	Bechati	188/215	190/205	180/214	162/162
F260	Pig	Bechati	188/215	183/205	180/214	162/186
F261	Pig	Bechati	188/215	183/205	180/180	162/186
F263 a	Pig	Bechati	188/215	183/205	180/214	162/186/190/218
F264	Pig	Bechati	188/215	183/205	180/180	162/180
F265	Pig	Bechati	188/215	183/205	180/214	162/186
F270 a	Pig	Bechati	188/215	183/205	180/214	162/186/208/233
F271	Pig	Bechati	188/215	183/205	180/180	162/186
F272 a	Pig	Bechati	188/215	183/205	214/214	162/186/218
F273 a	Pig	Bechati	188/215	183/205	180/180	162/186/190/218
F279	Pig	Bechati	188/215	183/205	180/180	162/186
F282 a	Pig	Bechati	138/188/215	183/205	180/180	162/186
F542 a	Pig	Bechati	188/215	183/205/240	180/214	162/180/190
F543	Pig	Bechati	188/215	183/205	180/214	162/186
F568 a,b	Pig	Bechati	188/215	136/190/240	180/214	000/000
F570	Pig	Bechati	190/215	190/190	180/180	169/180
F212	Goat	Besali	188/215	183/205	180/214	162/186
F506	Goat	Besali	188/215	183/205	180/195	162/162
F217 b	Sheep	Besali	000/000	183/205	180/214	162/162
F218	Sheep	Besali	188/215	183/205	180/214	162/186
F220	Sheep	Besali	190/215	183/205	180/214	162/186
F222 a,b	Sheep	Besali	000/000	183/205	180/214	162/186/208/233
F235	Sheep	Besali	190/215	183/205	180/180	162/186
F520 b	Sheep	Besali	188/188	183/205	000/000	162/162
F521	Sheep	Besali	177/177	183/205	180/180	162/186
F529	Sheep	Besali	188/215	183/205	173/195	162/186
F209 a	Pig	Besali	188/215	190/217	180/214	162/180/190
F228 a	Pig	Besali	188/215	190/217	180/214	162/186/190/218
F229 a	Pig	Besali	188/215	190/217	180/180	162/208/233
F503	Pig	Besali	188/215	183/205	180/214	162/186
F504 a	Pig	Besali	188/215	183/205	180/214	162/186/190/218
F523	Pig	Besali	188/215	183/205	180/195	162/186
F524	Pig	Besali	188/215	183/205	180/180	162/162
F525	Pig	Besali	188/215	183/205	180/214	162/186
F526	Pig	Besali	188/215	183/205	180/214	162/186
F289	Dog	Folepi	188/215	183/205	180/180	162/186
F579 a	Dog	Folepi	173/188/240	183/205	180/214	162/186
F585	Sheep	Folepi	188/215	183/205	200/200	162/186
F286	Pig	Folepi	188/215	190/217	180/180	162/186
F290	Pig	Folepi	188/215	183/205	180/180	162/186
F298	Pig	Folepi	188/215	183/205	180/214	162/186
F301 a	Pig	Folepi	138/173/188/240	183/205	180/214	162/186
F571 a	Pig	Folepi	190/215	183/205/240	180/189	162/180
F573 a	Pig	Folepi	138/188/215	183/205	180/180	169/190
F574	Pig	Folepi	188/215	183/205	180/214	169/190
F575 a	Pig	Folepi	138/188/215	183/205	180/214	162/186
F576 a	Pig	Folepi	138/188/215	183/205	180/214	162/186
F577	Pig	Folepi	188/215	183/205	200/200	162/186
F578	Pig	Folepi	188/215	183/205	180/214	162/186
F581 a	Pig	Folepi	138/188/215	183/205	180/214	162/186
F584	Pig	Folepi	188/215	183/205	180/180	162/186
F311	Goat	Menji	194/220	183/205	180/214	162/186
F312	Goat	Menji	188/215	190/205	180/214	162/186
F612 a	Goat	Menji	188/215	194/217	180/214	162/186/208
F613 a	Goat	Menji	166/215/240	183/205	173/210	162/186
F313	Pig	Menji	188/188	183/205	180/180	162/186
F314	Pig	Menji	188/215	183/205	180/180	162/186
F315	Pig	Menji	188/215	183/205	180/180	162/186
F316 a	Pig	Menji	173/188/215	183/205	180/214	162/186
F317 a	Pig	Menji	173/188/215	183/205	180/214	162/186
F318	Pig	Menji	188/188	183/205	189/189	162/186
F319	Pig	Menji	198/240	183/205	180/180	162/186
F320	Pig	Menji	188/215	183/205	180/214	162/186
F322	Pig	Menji	188/215	183/205	180/189	162/186
F323	Pig	Menji	188/215	183/205	189/189	162/186
F324	Pig	Menji	190/220	183/205	180/180	162/186
F325 a	Pig	Menji	188/215	183/205/240	180/214	162/186
F326	Pig	Menji	188/215	183/205	180/214	162/186
F327 b	Pig	Menji	188/215	183/205	180/214	000/000
F328	Pig	Menji	188/215	183/205	180/214	162/186
F331 a	Pig	Menji	190/220	183/205	180/214	162/180/190
F332	Pig	Menji	188/215	183/205	180/214	162/186
F333 a	Pig	Menji	188/215	183/205/240	180/214	162/186
F338 b	Pig	Menji	188/215	183/205	180/195	000/000
F343	Pig	Menji	188/215	190/205	180/214	162/186
F346	Pig	Menji	188/215	183/205	180/180	162/186
F348	Pig	Menji	188/215	183/205	180/214	162/186
F349	Pig	Menji	188/215	183/205	180/214	162/162
F604	Pig	Menji	188/215	183/205	180/214	162/186
F606	Pig	Menji	188/215	183/205	180/214	162/186
F608	Pig	Menji	188/215	194/217	173/210	162/186
F609	Pig	Menji	190/220	183/205	180/214	162/186
F611 a	Pig	Menji	190/220	194/217	180/214	162/186/190/218
F615	Pig	Menji	188/215	183/205	180/214	162/186
F617	Pig	Menji	188/215	183/205	180/214	162/186
F624	Pig	Menji	188/215	183/205	180/214	162/186
F625	Pig	Menji	188/215	183/205	180/214	162/186
F626	Pig	Menji	188/215	183/205	180/214	162/186
F627	Pig	Menji	188/215	183/205	180/214	162/186
F630	Pig	Menji	188/215	183/205	173/210	162/186
F632	Pig	Menji	188/215	183/205	173/173	162/186
F634	Pig	Menji	188/215	183/205	173/210	162/186
F636	Pig	Menji	188/215	183/183	173/210	162/162
F637	Pig	Menji	188/215	183/205	180/214	162/186
F638 a	Pig	Menji	188/215	183/205/240	180/214	162/186
F639 a	Pig	Menji	188/215	183/205	180/214	162/180/190
F640 a	Pig	Menji	188/215	190/205/240	180/214	162/186/218
F642	Pig	Menji	188/215	183/205	180/214	162/186
F644 a	Pig	Menji	188/215	183/205/225	180/214	162/186
F645	Pig	Menji	188/215	183/205	180/214	162/186
F646	Pig	Menji	188/215	183/225	180/214	162/186
F648	Pig	Menji	188/215	183/205	180/214	162/186
490	G. pal	Agong	188/215	183/205	180/214	162/186
177	G. pal	Bechati	188/215	183/205	180/214	162/186
145	G. pal	Bechati	188/215	183/205	180/214	162/186
124	G. pal	Bechati	194/194	205/240	180/214	162/186
120	G. pal	Bechati	188/215	183/205	180/214	162/186
123	G. pal	Bechati	188/215	183/205	180/214	162/186
2315	G. pal	Bechati	188/215	183/205	173/214	162/186
2264	G. pal	Bechati	188/215	183/205	180/214	162/186
2310	G. pal	Bechati	188/215	183/205	180/180	162/162
2279	G. pal	Bechati	188/215	183/205	180/214	162/186
2306	G. pal	Bechati	188/188	183/205	180/180	162/186
2202 a	G. pal	Bechati	188/215	183/205	173/173	162/186/208
2262 b	G. pal	Bechati	000/000	183/205	180/214	162/186
443	G. pal	Folepi	188/188	183/205	180/214	162/186
218	G. pal	Folepi	188/215	183/205	180/180	162/186
316 a	G. pal	Folepi	166/188/215	183/205	180/214	162/186
257	G. pal	Folepi	188/215	183/205	180/214	162/186
295	G. pal	Folepi	188/215	183/205	180/214	162/186
323 b	G. pal	Folepi	188/215	205/205	180/214	000/000
1116	G. pal	Folepi	188/215	183/205	180/214	162/186
1653	G. pal	Folepi	188/215	183/205	180/214	162/186
1835	G. pal	Folepi	188/215	183/205	180/214	162/186
1537	G. pal	Folepi	188/215	183/205	180/180	162/186
1129	G. pal	Folepi	188/215	183/205	180/214	162/186
1450	G. pal	Folepi	188/215	183/205	180/214	162/186
1506	G. pal	Folepi	188/215	183/183	185/185	162/186
1631	G. pal	Folepi	166/188	183/205	180/180	162/186
1657	G. pal	Folepi	188/215	183/205	180/214	162/186
2037	G. pal	Folepi	188/215	183/205	180/180	162/162
1119	G. pal	Folepi	188/188	183/205	180/214	162/186
1905	G. pal	Folepi	188/188	183/183	180/180	162/186
1680	G. pal	Folepi	188/215	183/205	180/180	162/162
1002	G. pal	Folepi	188/215	183/205	180/214	162/162
1970	G. pal	Folepi	188/215	183/205	180/214	162/186
1406	G. pal	Folepi	188/215	194/217	180/214	186/186
2031	G. pal	Folepi	188/215	183/205	180/214	162/186
1340	G. pal	Folepi	194/194	183/205	180/214	162/186
1075 a	G. pal	Folepi	166/194/215	183/205	180/214	162/186
2049	G. pal	Folepi	188/215	183/205	180/214	162/186
1823	G. pal	Folepi	188/215	183/205	180/214	162/186
1602	G. pal	Folepi	188/215	183/205	180/214	162/162
2029	G. pal	Folepi	188/188	183/205	180/214	162/186
1677	G. pal	Folepi	188/215	183/205	180/214	162/186
1587 b	G. pal	Folepi	194/194	183/183	000/000	000/000
2042 b	G. pal	Folepi	188/215	000/000	180/180	169/208
1228 ^b^	G. pal	Folepi	000/000	194/217	180/214	000/000
1415 b	G. pal	Folepi	188/215	000/000	180/214	000/000
2446	G. pal	Menji	188/188	183/205	180/180	162/186
2471	G. pal	Menji	188/215	194/217	180/214	162/186
2542	G. pal	Menji	194/194	183/205	180/214	162/186
2537 b	G. pal	Menji	188/215	183/205	000/000	000/000
2549 b	G. pal	Menji	000/000	183/205	180/214	000/000
2531	G. pal	Menji	188/215	183/205	180/214	162/186

a : sample has multiple infections

b : sample amplified at less than 5 loci
